# 

**Published:** 1887-10

**Authors:** 


					﻿Vol VII.
OCTOBER, 1887.
No. 10.
THE
HOMEOPATHIC pHY£lClAJI,
A MONTHLY JOURNAL OF MEDICAL SCIENCE.
"He is a freeman whom truth makes free, and all are slaves beside. ”
"Seek the Truth: come whence it may, cost what it will.”
EDITED BY
Edmund j. lee, m. d.,
AND
WALTER M. JAMES, M. D.
PHILADELPHIA :
No. 1123 SPRUCE STREET.
STEW YORK
A. L. CHATTERTON & COMPANY, Publisher’s Agents.
LONDON:
ALFRED HEATH A CO., Sole Agents fob Gbeat Britain,
114 Ebury Street, Eaton Square.
Yearly Subscription, $2.SO, invariably in advance. Single numbers, 95 cte.
Entered at the Philadelphia Poet-Office an Second-Claae Mail Matter.
				

